# Limb composition: implications for the determination of arterial occlusion pressure

**DOI:** 10.3389/fphys.2026.1707774

**Published:** 2026-05-29

**Authors:** Júlio M. Alves, Victor S. de Queiros, Nicholas Rolnick, Rúben Arnaldo de Oliveira-Silva, Isabelle Feitosa Laureano, Vanessa Carla Monteiro Pinto, José Damião Rodrigues, Breno Guilherme de Araújo Tinôco Cabral, Paulo Moreira Silva Dantas

**Affiliations:** 1 Graduate Program in Health Sciences, Federal University of Rio Grande do Norte, Natal, Brazil; 2 Department of Physical Education, State University of Paraíba, Campina Grande, Brazil; 3 The BFR PROS, New York, NY, United States; 4 PHITWELL, New York, NY, United States; 5 Department of Physical Education, Federal University of Rio Grande do Norte, Natal, Brazil

**Keywords:** blood flow restriction therapy, blood pressure, body composition, exercise prescription, limb arterial occlusion

## Abstract

**Background:**

Blood flow restriction (BFR) training involves exercising under partial vascular occlusion to induce muscular adaptations, and the accurate determination of arterial occlusion pressure (AOP) is essential for its safe and effective prescription. Although limb size and blood pressure are established determinants of AOP, the role of lower-limb tissue composition and the influence of body position and sex on these relationships remain poorly understood.

**Purpose:**

This study aimed to examine the association between lower-limb body composition and lower-limb AOP measured using an 18-cm cuff across different body positions, while also exploring the influence of sex.

**Methods:**

Fifty-one healthy adults (25 men, 26 women) participated in this cross-sectional study. Whole-body and lower-limb composition were assessed using dual-energy X-ray absorptiometry (DXA). AOP was measured at the right thigh using a portable Doppler ultrasound in supine, seated, and standing positions. Associations were examined using bivariate correlations, multiple regression models adjusted for sex, commonality analyses (LMG metric), and linear mixed models for interactions.

**Results:**

In the seated position, fat mass was positively associated with AOP (β = 3.55; 95% CI: 0.15 to 6.96; p = 0.041), whereas lean mass was not. In the standing position, both lean mass (β = 2.96; 95% CI: 1.10 to 4.82; p = 0.002) and fat mass (β = 3.44; 95% CI: 0.48 to 6.40; p = 0.023) were positively associated with AOP. A significant lean mass × position interaction indicated weaker associations in the seated (β = −1.61; p < 0.001) and supine (β = −1.90; p < 0.001) positions compared with standing, while a significant fat mass × position interaction was observed only between the seated and supine positions (β = −2.45; p = 0.031). No significant sex-related interactions were detected.

**Conclusion:**

These findings indicate that lower-limb composition, especially lean and fat mass, is associated with AOP in a position-dependent manner, suggesting that tissue characteristics may be considered when estimating occlusion pressure using indirect methods.

## 1 Introduction

Blood flow restriction (BFR) training involves performing exercises while applying restriction to the active limb, with the aim of promoting muscular adaptations such as hypertrophy and strength gains using reduced loads (e.g., 20–40% of one-repetition maximum) (Laurentino et al., 2012; Lixandrão et al., 2015). BFR can be applied using various devices, such as elastic bands or inflatable cuffs (Freitas et al., 2020), to partially restrict arterial inflow and occlude venous return (Patterson et al., 2019). This restriction promotes the production and accumulation of metabolites in the active muscle, which is believed to accelerate fatigue and stimulate the recruitment of type II muscle fibers (Loenneke et al., 2010).

In this method, cuff pressure should occlude venous return while only partially restricting arterial inflow. Despite the use of arbitrary pressures, it is recommended that pressure be individualized as a percentage of arterial occlusion pressure (AOP) (Patterson et al., 2019), which is defined as the minimum pressure required to completely interrupt arterial blood flow. AOP can be influenced by several factors, including body position, cuff placement and width, and anthropometric characteristics (de Queiros et al., 2024a). In this context, limb circumference has consistently demonstrated a strong positive association with AOP, particularly when narrower cuffs are used. This relationship is likely attributable to the greater volume of tissue surrounding the vasculature in larger limbs, which increases the pressure required to collapse the artery (Jessee et al., 2016; Loenneke et al., 2012, Loenneke et al., 2015). As a result, this variable has been commonly included in predictive equations for AOP (Loenneke et al., 2012; Wedig et al., 2024; Sieljacks et al., 2018).

Beyond simple anthropometric measures, the influence of limb composition on AOP has been explored in a limited number of studies. Loenneke et al (Loenneke et al., 2015). reported that a predictive model incorporating brachial systolic blood pressure (SBP) together with muscle and adipose tissue thickness explained a greater proportion of the variance in upper-limb AOP than a model based solely on SBP (58% vs. 51%, respectively), with positive associations observed between tissue thickness and AOP. Similarly, two previous studies demonstrated that both muscle cross-sectional area (mCSA) and fat cross-sectional area (fCSA), assessed by computed tomography or predictive equations, were positively associated with lower-limb AOP. Wedig et al (Wedig et al., 2024). indicated that mCSA is a good predictor of lower-limb AOP when using 18-cm wide cuff. Additionally, Loenneke et al (Loenneke et al., 2012). reported significant associations of limb composition with AOP, with fCSA explaining the largest proportion of variance, regardless of whether AOP was determined using a 13.5-cm or a 5-cm cuff. These findings suggest that soft tissue characteristics contribute meaningfully to the pressure required to occlude arterial blood flow.

In contrast, when analyzing the lower limbs, one study identified an inverse relationship between lean mass assessed by dual-energy X-ray absorptiometry (DXA) and AOP (Walden et al., 2024), a finding that diverges from the associations reported by previous studies (Loenneke et al., 2012, Loenneke et al., 2015; Wedig et al., 2024). Multiple factors may account for the divergence observed among findings in the literature, including the method used to evaluate limb composition, participants’ characteristics, and cuff width. For example, wider cuffs (as 18-cm cuffs) distribute external pressure over a larger surface area, thereby reducing the pressure required to occlude arterial flow. Consequently, the effect of cuff width on AOP may be attenuated (Crenshaw et al., 1988). The limited number of studies examining the relationship between limb composition variables and AOP restricts more definitive conclusions on this topic and highlights the need for further research to advance the field.

In addition to limb composition, sex has also been examined as a potential predictor of AOP, with conflicting findings. For example, Tafuna’i et al (Tafuna’i et al., 2021). reported that sex was no longer a significant predictor of lower-limb AOP after adjustment for limb circumference, suggesting that sex-related differences may be largely mediated by anthropometric characteristics. Conversely, Jessee et al (Jessee et al., 2016). identified sex as a significant predictor of AOP, indicating that differences in tissue composition or vascular properties may persist even after accounting for limb size. For example, differences in arterial stiffness and sympathetic activation may influence the pressure required to achieve AOP (DuPont et al., 2019; Jacob et al., 2021). Additionally, differences in limb tissue composition may affect the transmission of pressure to the vasculature.

Finally, body position may further modify the relationship between limb composition and AOP, particularly in the lower limbs. Changes in body position alter hydrostatic pressure, venous pooling, and baseline muscle tone, which may influence arterial transmural pressure and the effectiveness of external compression. Previous studies have investigated this relationship only in the supine position (Loenneke et al., 2012, Loenneke et al., 2015; Walden et al., 2024) and in the seated position (Wedig et al., 2024), which may partly explain divergent findings across studies. Despite its physiological relevance, the extent to which body position interacts with anthropometric and compositional variables to determine AOP remains poorly understood.

Therefore, the aim of this study was to examine the association between lower-limb body composition and lower-limb AOP measured using an 18-cm cuff across different body positions, while also exploring the influence of sex. Based on the aforementioned considerations, we hypothesized that limb composition would be associated with AOP, and that sex and body position would significantly influence this association.

## 2 Materials and methods

### 2.1 Study design and participants

This cross-sectional observational study analyzed secondary data from a previous investigation (de Queiros et al., 2024b) to address a new research question. Data were obtained from 51 healthy participants (25 men and 26 women) with no known cardiovascular, metabolic, or musculoskeletal disorders.

### 2.2 Ethical approval

The study was approved by the institutional ethics committee of the Federal University of Rio Grande do Norte (Approval number: 6,599,200) and conducted in accordance with the Declaration of Helsinki. All participants provided written informed consent prior to participation.

### 2.3 Procedures

During a single laboratory visit, all participants underwent anthropometric and peripheral vascular measures assessments. Anthropometric measurements included height, body mass, and thigh circumference, which was measured on the right thigh at a distance corresponding to 33% of the length from the inguinal fold to the top of the patella (Loenneke et al., 2015; Sieljacks et al., 2018). Thigh circumference was measured by a single evaluator three times, and the mean value was used for analysis (Norton, 2018). Peripheral vascular measures assessments included AOP and brachial blood pressure, with participant characteristics summarized in **Table 1**. Assessments were conducted in the afternoon (2:00 p.m. to 4:30 p.m.) in a controlled environment with temperature maintained between 21 °C and 24 °C. Participants were instructed to abstain from caffeine and strenuous exercise and to maintain their usual water intake on the day of the assessment.

Upon arrival, participants rested for 10 minutes before brachial blood pressure was measured using an automatic blood pressure monitor (Omron^®^, HEM7200, Omron, USA) in the supine position. Two measurements were taken at 1-minute intervals. When the difference between measurements was ≥5 mmHg, a third measurement was performed. is, the mean of the two closest values was used (Loenneke et al., 2012).

After an additional 3-minute rest period, AOP was assessed by a single evaluator in a randomized order across three positions (supine, seated, and standing), with a 5-minute interval between measurements. A nylon cuff (width = 18 cm, length = 75 cm; Premium^®^, Brazil) was placed on the proximal portion of the right thigh, and a portable vascular Doppler (Medpej^®^, DF7001-VN; 8-MHz; ultrasonic power: <5 mW/cm^2^) was positioned over the posterior tibial artery. The cuff was gradually inflated in 20 mmHg increments until the arterial pulse ceased, as indicated by the absence of audible signals on the Doppler. Immediately thereafter, the cuff was inflated by an additional 20 mmHg and then slowly deflated to confirm the observed AOP value (Cirilo-Sousa et al., 2019). AOP was measured once in each position, and the obtained value was used for analysis. In the supine position, participants were positioned in the dorsal decubitus position, with the hips and knees extended. In the seated position, participants remained seated on a chair with their knees and hip flexed at 90°. In the standing position, participants were instructed to maintain their weight evenly distributed between both legs. Additional procedural details can be found in a previous study (de Queiros et al., 2024b).

#### 2.3.1 Body composition assessment

Body composition was assessed using dual-energy X-ray absorptiometry (DXA) (LUNAR^®^/GE PRODIGY – LNR 41,990, Washington, DC, United States; software: enCORE, GE Healthcare^®^, version 15.0, Madison, WI, USA). The assessments were performed using the following device settings: whole-body scan mode, voltage (kV): 76.0, current (mA): 0.150, and radiation dose (µGy): 0.4 (very low, with no health risk). The analyzed variables included total body fat percentage, lower-limb fat percentage, total lower-limb mass, lower-limb lean mass, and lower-limb fat mass. Lower-limb composition was defined according to the standard regional boundaries provided by the software. The precision of the analyses performed with a Lunar DPX-L instrument (similar to the Prodigy, according to the manufacturer), established by repeated measurements over four consecutive days, was 1% for fat-free mass and 2% for fat mass (Kiebzak et al., 2000; Williams et al., 2006).

### 2.4 Statistical analysis

Because this study is a secondary analysis of a previous investigation, it was not possible to perform an *a priori* power analysis. Therefore, *post hoc* power analyses were therefore conducted (α = 0.05; n = 51) to assess the probability of detecting the observed effects.

For the multiple regression models that investigated both the associations between limb composition variables and AOP, as well as the unique and shared contributions of limb composition, limb circumference, SBP, and sex to AOP, statistical power was estimated based on the model’s coefficient of determination (R^2^). Effect size was expressed as Cohen’s f^2^, calculated from the R^2^ of the full model (f^2^ = R^2^/[1 − R^2^]). Statistical power was then computed using the F-test for multiple regression via the pwr.f2.test function from the pwr package (Champely, 2006; Cohen, 2013).

For the linear mixed-effects models used to investigate interactions between limb composition variables and body position or sex, statistical power was assessed using simulation-based approaches comparing nested models. Models with and without the interaction term were compared using likelihood ratio tests, with 1,000 simulations, implemented through the simr package (Green and MacLeod, 2016).

Data normality was assessed using the Shapiro–Wilk test and Q–Q plots. Sex differences in participant characteristics were analyzed using independent-samples Student’s t-tests (for parametric data) or Mann–Whitney U tests (for nonparametric data). Effect size was expressed as Cohen’s d for parametric comparisons and rank-biserial correlation for non-parametric comparisons. Pearson’s correlation test was used to evaluate bivariate associations between body composition variables (total body fat percentage, lower-limb fat percentage, total fat mass, and total lean mass) and AOP in each body position. Subsequently, variables that showed significant correlations were entered into multiple regression models to examine the association between limb composition and AOP. All models were adjusted for sex to control for potential sex-related differences in tissue properties and vascular responses that may not be fully captured by limb composition. Scatter plots were constructed to illustrate the significant associations between lower-limb composition and AOP, with data points differentiated by sex.

To examine the predictive value of limb composition relative to other predictors established in previous studies, commonality analysis was performed using the LMG metric. This approach decomposed the explained variance (R^2^) into the unique and shared contributions of each variable.

Linear mixed-effects models were used to investigate whether the relationship between lower-limb composition and AOP differed according to sex or body position. Participant ID was included as a random intercept to account for repeated AOP measurements across different body positions. Interaction terms between limb composition variables (lean mass and fat mass) and sex, as well as between limb composition variables and body position, were tested. These models were used exclusively to assess effect modification rather than to evaluate the main effects of sex or position.

Model assumptions were evaluated through inspection of residual plots, assessment of residual normality, and examination of homoscedasticity. Multicollinearity was defined as VIF ≥ 5 and/or Pearson r ≥ 0.80 (Snee, 1977) and was assessed using variance inflation factors and pairwise correlation coefficients. All statistical analyses were performed using R software (Version 4.5.2; R Foundation for Statistical Computing, Vienna, Austria), with the relaimpo package used for commonality analyses and the lme4 and lmerTest packages for linear mixed-effects models (Grömping, 2006; Bates et al., 2015; Kuznetsova et al., 2017). Statistical significance was set at p < 0.05.

## 3 Results

Participant characteristics are exhibited in **Table 1**. Variables with a normal distribution are expressed as mean and standard deviation, whereas non-normally distributed variables are demonstrated as median and 25th–75th percentiles. In the comparison between sexes, height, body weight, body mass index (BMI), AOP in the standing position, and lower-limb lean mass was significantly higher in men, whereas fat mass variables were significantly higher in women (**Table 1**). Additional interpretations regarding participant characteristics are discussed in a previous study (de Queiros et al., 2024b).

**Table 1:** Participants characteristics.

Variable	Total	Males	Females	*p-value*	*Effect size*
Age (years)	24.0 (22.0-27.0)	24 (21.5-26.5)	25 (22.75–29.0)	0.142	0.232
Height (cm)	167.9 (10.3)	175.8 (7.4)	160.4 (6.4)	**<0.001**	**2.228**
Body mass (kg)	68.09 (13.4)	83 (65.25–86.6)	59.2 (8.3)	**<0.001**	**1.830**
BMI (kg/m^2^)	24.6 (21.4–26.4)	25.4 (22.4–27.4)	23.0 (20.9-25.7)	**0.014**	**-0.400**
Thigh Circumference (cm)	59.2 (52.6–61.0)	59.7 (52.1–63.0)	57.9 (53.5–59.7)	0.070	0.438
Total body fat (%)	27.8 (8.1)	21.9 (5.8)	34.5 (31.1-37.6)	**<0.001**	**-2.056**
Lower limbs fat (%)	29.1 (8.5)	22.2 (5.1)	35.8 (5.0)	**<0.001**	**-2.697**
Lower limbs total mass (kg)	24.5 (4.9)	27.3 (4.8)	21.8 (3.3)	**<0.001**	**1.340**
Lower limbs fat mass (kg)	6.7 (2.0)	5.8 (1.9)	7.5 (1.7)	**0.002**	**-0.937**
Lower limbs lean mass (kg)	16.8 (4.5)	20.2 (3.6)	13.5 (2.1)	**<0.001**	**2.339**
AOP – Supine position (mmHg)	126.0 (14.5)	130.0 (12.1)	123.0 (15.9)	0.085	0.493
AOP – Seated position (mmHg)	164.0 (21.3)	166.0 (19.0)	162.0 (23.5)	0.444	0.216
AOP – Standing position (mmHg)	178.6 (22.3)	186.6 (23.0)	170.9 (19.0)	**0.010**	**0.748**

The values of the variables are expressed as mean (SD) and median (25th–75th percentiles) for normal and non-normal variables, respectively. The p-values and effect sizes represent comparisons between the sexes. BMI, body mass index; AOP, arterial occlusion pressure; mmHg, millimeters of mercury. Bold values represent statistical significance.

In the bivariate analyses (**Table 2**), lean mass showed a significant correlation with AOP in the supine (r = 0.317; p = 0.023) and standing positions (r = 0.586; p < 0.001), whereas the other variables did not demonstrate significant associations (p > 0.05). In the seated position, only fat mass showed a significant correlation (r = 0.286; p = 0.041), while the association with lean mass showed a trend toward significance (r = 0.274; p = 0.051).

**Table 2:** Pearson’s correlation coefficients between body composition variables and AOP in different body positions.

Variable	r	p value
Supine position		
Body fat %	0.009	0.946
Leg fat %	-0.111	0.434
Fat mass (kg)	0.083	0.558
Lean mass (kg)	0.317	**0.023**
Seated position		
Body fat %	0.149	0.296
Leg fat %	0.047	0.741
Fat mass (kg)	0.286	**0.041**
Lean mass (kg)	0.274	0.051
Standing position		
Body fat %	-0.119	0.404
Leg fat %	-0.200	0.158
Fat mass (kg)	0.228	0.106
Lean mass (kg)	0.586	**< 0.001**

kg, kilogram. Bold values represent statistical significance.

Based on these findings, the variables that demonstrated bivariate associations with AOP (lean mass and fat mass) were selected for inclusion in the subsequent multivariable analyses.

### 3.1 Lower limb composition and arterial occlusion pressure

**Figure 1:**
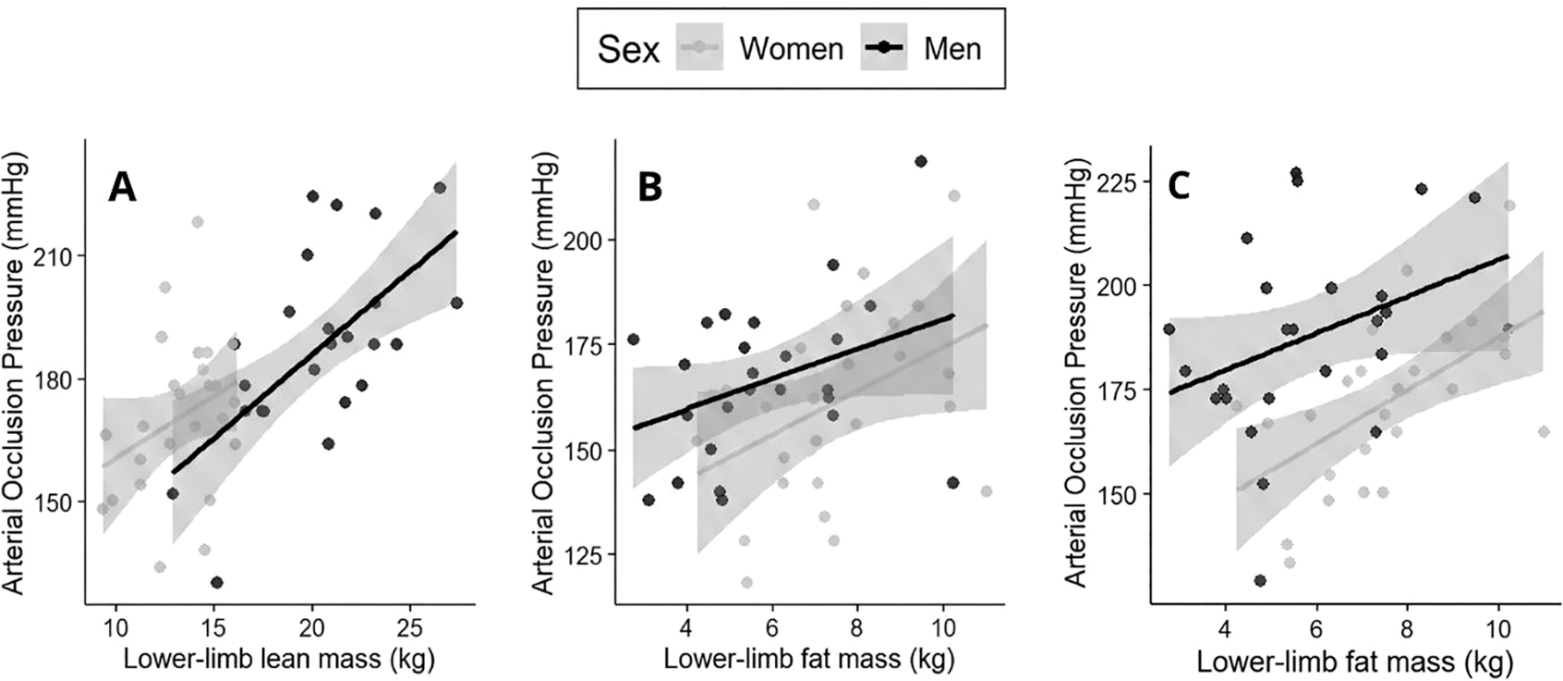
Scatter plots illustrating the significant associations between lower-limb composition and arterial occlusion pressure (AOP) across different body positions. **(A)** Relationship between lower-limb lean mass and AOP in the standing position; **(B)** relationship between lower-limb fat mass and AOP in the seated position; and **(C)** relationship between lower-limb fat mass and AOP in the standing position. Grey points and lines represent women, while black points and lines represent men. Shaded areas indicate the 95% confidence intervals.

The associations between AOP and limb composition variables, adjusted for sex, are presented in **Table 3**. In the supine position, none of the limb composition variables showed a significant association with AOP (p > 0.05; r^2^ = 0.121). In the seated position (r^2^ = 0.177), lower-limb fat mass was positively associated with AOP (β = 3.55; 95% CI: 0.15 to 6.96; p = 0.041) (**Figure 1B**), whereas lean mass did not show a significant association. In the standing position (r^2^ = 0.431), both lean mass (**Figure 1A**) (β = 2.96; 95% CI: 1.10 to 4.82; p = 0.002) and fat mass (**Figure 1C**) (β = 3.44; 95% CI: 0.48 to 6.40; p = 0.023) were positively and significantly associated with AOP. Sex was not significantly associated with AOP in any of the positions analyzed.

**Table 3:** Associations between limb composition and arterial occlusion pressure in different body positions.

Variable	β (CI 95%)	Stand. β (CI 95%)	Stand. error	p value
Supine position				
Sex	-4.17 (-18.78; 10.44)	-0.29 (-1.30; 0.72)	7.26	0.569
Lean mass (kg)	0.73 (-0.12; 3.18)	0.22 (-0.24; 0.69)	0.74	0.335
Fat mass (kg)	1.24 (-1.15; 3.63)	0.17 (-0.16; 0.50)	1.19	0.301
r^2^ = 0.121				
Seated position				
Sex	-1.51 (-22.34; 19.32)	-0.07 (-1.05; 0.91)	10.35	0.885
Lean mass (kg)	1.35 (-0.78; 3.49)	0.28 (-0.16; 0.73)	1.06	0.209
Fat mass (kg)	3.55 (0.15; 6.96)	0.33 (0.01; 0.65)	1.69	**0.041**
r^2^ = 0.177				
Standing position				
Sex	-1.47 (-19.58; 16.62)	-0.07 (-0.88; 0.74)	9.00	0.870
Lean mass (kg)	2.96 (1.10; 4.82)	0.59 (0.22; 0.97)	0.92	**0.002**
Fat mass (kg)	3.44 (0.48; 6.40)	0.31 (0.04; 0.57)	1.47	**0.023**
r^2^ = 0.431				

CI 95%, confidence interval of 95%; kg, kilogram; Stand. β, standard β; Stand. error, standard error. Bold values represent statistical significance.

### 3.2 Relative contribution of circumference, limb composition, sex, and systolic blood pressure

**Table 4** shows the coefficients of the multivariable models that included lower-limb composition, thigh circumference, SBP, and sex, as well as the results of the commonality analysis.

**Table 4:** Multivariable associations and relative contribution of limb composition, circumference, and systolic blood pressure to arterial occlusion pressure in different body positions.

Variable	Coefficients of the model				Commonality analysis	
	β (CI 95%)	Stand. β (CI 95%)	Stand. error	p value	Unique r^2^	% of total r^2^
Supine position						
Lean mass (kg)	-1.26 (-3.40, 0.88)	-0.39 (-1.05; 0.27)	1.06	0.241	0.036	0.087
Fat mass (kg)	0.04 (-2.47, 2.54)	0.00 (-0.34; 0.35)	1.25	0.978	0.010	0.024
Thigh Circumference	1.17 (-0.33, 2.67)	0.41 (-0.12; 0.94)	0.74	0.123	0.074	0.179
SBP	0.72 (0.36, 1.08)	0.54 (0.27; 0.81)	0.18	**< 0.001**	0.270	0.650
Sex	-6.34 (-20.18, 7.50)	-0.44 (-1.40; 0.52)	6.87	0.360	0.025	0.060
r^2^ = 0.415						
Seated position						
Lean mass (kg)	-0.64 (-3.74, 2.46)	-0.13 (-0.78; 0.51)	1.54	0.679	0.029	0.067
Fat mass (kg)	2.59 (-1.05, 6.22)	0.24 (-0.10; 0.58)	1.80	0.159	0.060	0.138
Thigh Circumference	0.93 (-1.25, 3.10)	0.22 (-0.30; 0.74)	1.08	0.395	0.085	0.196
SBP	1.07 (0.55, 1.59)	0.55 (0.28; 0.81)	0.26	**< 0.001**	0.249	0.572
Sex	-1.41 (-21.45, 18.64)	-0.07 (-1.01; 0.87)	9.95	0.888	0.012	0.027
r^2^ = 0.435						
Standing position						
Lean mass (kg)	1.76 (-0.94, 4.46)	0.35 (-0.19; 0.89)	1.34	0.195	0.132	0.217
Fat mass (kg)	3.07 (-0.08, 6.24)	0.27 (-0.01; 0.56)	1.57	0.056	0.053	0.088
Thigh Circumference	0.33 (-1.56, 2.22)	0.08 (-0.36; 0.51)	0.94	0.725	0.140	0.231
SBP	0.97 (0.51, 1.42)	0.47 (0.25; 0.69)	0.22	**< 0.001**	0.234	0.384
Sex	0.68 (-16.77, 18.13)	0.03 (-0.75; 0.81)	8.66	0.938	0.049	0.080
r^2^ = 0.608						

CI 95%, confidence interval of 95%, kg, kilogram, SBP, systolic blood pressure, Stand. β, standard β; Stand. error, standard error. Bold values represent statistical significance.

Across all body positions, SBP was the strongest individual predictor of AOP, showing a significant association (p < 0.001) and the largest unique and relative contribution to the total r^2^ of the models (ranging from 38.4% to 65.0%).

Thigh circumference made a relevant contribution to the explained variance in AOP, although its individual associations did not reach statistical significance in any of the positions. In contrast, lower-limb composition variables showed smaller and position-dependent contributions. When combined, lean mass and fat mass contributed less in the supine position (unique r^2^ = 0.046 vs. unique r^2^ = 0.074), similarly in the seated position (unique r^2^ = 0.089 vs. unique r^2^ = 0.085), and more in the standing position (unique r^2^ = 0.185 vs. unique r^2^ = 0.140), compared with thigh circumference. Between the two, lean mass showed the greatest unique contribution among the composition variables in the standing (unique r^2^ = 0.132; %r^2^ = 21.7%) and supine positions (unique r^2^ = 0.036; % r^2^ = 8.7%), whereas in the seated position, fat mass (unique r^2^ = 0.060; % r^2^ = 13.8%) showed the greater relative contribution.

Sex showed a limited contribution to the explained variance in AOP across all positions.

### 3.3 Interactions between body position, sex, and lower-limb composition in their relationships with arterial occlusion pressure

In the interaction analyses (**Table 5**), significant interactions were observed between lower-limb composition and body position. Specifically, the association between lean mass and AOP was significantly weaker in the seated (β = −1.61; p < 0.001) and supine (β = −1.90; p < 0.001) positions compared with the standing position, indicating that the relationship between lean mass and AOP is position-dependent. Regarding fat mass and AOP, a significant difference was observed only between the seated and supine positions, with a stronger association in the seated position. (β = −2.45; p = 0.031).

**Table 5:** Interactions between limb composition, body position, and sex in predicting arterial occlusion pressure.

Interaction	β (CI 95%)	Stand. β (CI 95%)	Stand. error	p value
Body position				
Lean mass (kg) Standing x Seated position	-1.61 (-2.52; -0.70)	-0.24 (-0.38; -0.10)	0.465	**< 0.001**
Lean mass (kg) Standing x Supine position	-1.90 (-2.81; -0.99)	-0.28 (-0.42; -0.15)	0.465	**< 0.001**
Lean mass (kg) Seated x Supine position	-0.28 (-1.21; 0.65)	-0.04 (-0.18; 0.10)	0.474	0.554
Fat mass (kg) Standing x Seated position	0.51 (-1.66; 2.68)	0.03 (-0.12; 0.18)	1.105	0.647
Fat mass (kg) Standing x Supine position	-1.94 (-4.11; 0.23)	-0.13 (-0.28; 0.02)	1.105	0.082
Fat mass (kg) Seated x Supine position	-2.45 (-4.66; -0.24)	-0.16 (-0.31; -0.01)	1.13	**0.032**
Sex				
Lean mass (kg) x Sex	0.94 (-1.09; 3.85)	0.14 (-0.28; 0.57)	1.429	0.512
Fat mass (kg) x Sex	-0.75 (-4.47; 2.98)	-0.05 (-0.30; 0.20)	1.899	0.696

CI 95%, confidence interval of 95%; kg, kilogram; Stand. β, standard β; Stand. error, standard error. Bold values represent statistical significance.

Otherwise, no significant interactions were identified between sex and either lower-limb lean mass or fat mass in the prediction of AOP, suggesting that the relationship between limb composition and AOP does not differ between men and women after statistical control for tissue-related variables. However, the absence of significant sex-related interactions should be interpreted with caution, as this analysis had limited power to detect small interaction effects (Power = 0.070 and 0.116).

## 4 Discussion

The aim of this study was to examine the association between lower-limb body composition and lower-limb AOP, measured using an 18-cm cuff in different body positions, while also exploring the influence of sex. As main findings, we identified that, in a model including sex, lean mass (kg), and fat mass (kg), limb composition variables were positively associated with lower-limb AOP measured in the standing position. In the seated position, only lower-limbs fat mass was associated with AOP, whereas no significant associations were identified in the supine position. However, after including established predictors such as brachial SBP and thigh circumference, these associations were no longer statistically significant. In addition, SBP explained the greatest proportion of the AOP variance, and limb composition contributed more to the explained variance than thigh circumference only in the standing position.

### 4.1 Influence of lower limb composition on arterial occlusion pressure

Our results partially differ from those reported by Loenneke et al (Loenneke et al., 2012). who identified body composition variables assessed by computed tomography as important predictors of lower-limb AOP in the supine position, particularly fCSA, in models that included brachial systolic and diastolic blood pressure. Some methodological differences may contribute to these discrepancies, most notably the cuff width used (5 and 13.5 cm versus 18 cm). Recent evidence suggests that the magnitude of the contribution of AOP predictors may be influenced by cuff width (de Queiros et al., 2024b; Wedig et al., 2025). Specifically, it has been observed that the proportion of variance in AOP explained by thigh circumference tends to decrease as cuff width increases. Thus, it is possible that the influence of limb composition on AOP is attenuated when measurements are performed using wider cuffs.

While the influence of thigh composition on AOP is reduced with the use of wider cuffs, the contribution of SBP appears to be amplified. It is possible that the greater capacity for force transmission through the tissues provided by wider cuffs attenuates the influence of tissue characteristics, whereas intravascular pressure becomes one of the main variables explaining the variation in AOP. This may explain our findings, which demonstrated that SBP explained a larger proportion of the variance in AOP when included in the models.

When considering only limb composition and sex, both fat mass and lean mass were positively associated with AOP, particularly in the standing position. Adipose tissue is more compliant and deformable, which may have absorbed part of the force exerted by the cuff, thereby reducing its effectiveness in compressing the blood vessels. In contrast, skeletal muscle—the primary component of lean mass—is mechanically stiffer (Böl et al., 2014), which may facilitate more efficient transmission of the applied force to the vasculature, potentially resulting in lower AOP in individuals with greater muscle mass. However, in the present study, AOP was positively associated with lean mass, consistent with previous studies examining its relationship with muscle mass (Loenneke et al., 2012, 2015; Wedig et al., 2024). It is possible that, despite its mechanical properties favoring pressure transmission from the cuff to the vasculature, the contribution of lean mass and muscle mass to overall limb size remains a predominant factor underlying its association with AOP.

In contrast, Walden et al (Walden et al., 2024). reported a negative association between muscle mass and AOP, suggesting that greater amounts of lower-limb muscle mass would be associated with a lower pressure required to occlude blood flow. These findings contradict both the present study and previous investigations that have identified a positive association between muscle mass (or, in our case, lean mass) and AOP (Loenneke et al., 2012, Loenneke et al., 2015; Wedig et al., 2024). This discrepancy may be explained by methodological differences. Unlike the present study, Walden et al (Walden et al., 2024). partitioned DXA-derived lean mass into muscle mass and bone mass separately. Whether this partitioning was performed directly by the DXA software or estimated via predictive equations remains unclear; either way, this methodological difference may have contributed to the observed discrepancies. Additionally, the relatively small sample size may have resulted in unstable coefficients that are more susceptible to the influence of outliers, and the statistical analyses employed also differed between studies (partial Pearson correlations vs multiple variables modeling).

In another study, Yaşar et al (Yaşar et al., 2025). examined an exclusively male sample and investigated the relationship between AOP and anthropometric variables, including body fat percentage. The authors reported a statistically significant, albeit weak, correlation between AOP and body fat percentage (r = 0.149). However, in the present study, this body composition variable was not significantly associated with AOP in the correlation analyses and was therefore not included in the models. Despite these contrasting findings, the authors of the aforementioned study did not report the method used to assess body fat percentage, which limits the identification of potential explanations for these differences.

### 4.2 Role of body position

Among the factors influencing AOP, previous studies have highlighted the role of body position (Hughes et al., 2018; Sieljacks et al., 2018). Variations in AOP across positions are largely attributed to the effects of hydrostatic pressure in the lower limbs. One of these effects is fluid redistribution, which increases interstitial fluid volume in both muscle and adipose tissues. This, in turn, may increase limb volume and elevate resistance to cuff compression (Berg et al., 1993; Maw et al., 1995). In addition, in the standing position, lower-limb muscles such as the vastus lateralis remain continuously active to support body weight (Clarke et al., 2023), which may generate a force opposing cuff compression. These factors may contribute to a stronger association between lean tissue and AOP and to the differences observed between the standing position and the other postures.

Interestingly, a difference in the relationship between AOP and fat mass was observed between a low hydrostatic load condition (supine) and an intermediate hydrostatic load condition (seated), but not between the two extremes (low and high hydrostatic load). One possible explanation is that the more subtle changes in interstitial and vascular fluids between these two positions—relative to the transition from supine to standing—may have mediated this finding. Compared with the supine position, the seated position is characterized by greater interstitial pressure and volume, which may affect the compressibility of adipose tissue due to an increase in its volume (Maw et al., 1995; Husmann et al., 2006). However, under conditions of greater hydrostatic pressure—such as in the standing position—the influence of adipose tissue may be overshadowed by other factors, including higher baseline vascular pressure and sympathetic hemodynamic adjustments (Trybulski et al., 2025). Additionally, skeletal muscle in the compressed region is more actively recruited during standing (Clarke et al., 2023), which may increase its stiffness and modify the transmission of external pressure to the vasculature. However, this is speculative and should be tested in future studies.

### 4.3 Sex differences in lower-limb composition and arterial occlusion pressure

In previous studies, biological sex has been a controversial variable with respect to its influence on AOP prediction. Jessee et al (Jessee et al., 2016). found a significant contribution of sex to the explained variance in AOP measured with 5-cm, 10-cm, and 12-cm cuffs applied to the upper limbs, even after including limb circumference in the models. However, the magnitude of the sex-related differences in AOP was relatively small, which may not be clinically relevant for BFR exercise prescription. In contrast, Tafuna’i et al (Tafuna’i et al., 2021). reported that sex did not make a significant contribution to the prediction of AOP measured in the lower limbs using a 10-cm cuff when thigh circumference was included in the models. Similarly, our findings showed that sex did not contribute significantly when included in the same model as limb composition variables, although men exhibited higher AOP values than women.

Despite well-established sex-related differences in the distribution of body composition variables and in the structural characteristics of these tissues, these differences may not be sufficient to significantly influence AOP. The lower-limb composition of men and women tends to exhibit distinct patterns, particularly in the thigh region (Abe et al., 2003; Karastergiou et al., 2012). However, when these variables are accounted for in regression models, sex is no longer significantly associated with AOP, and the observed differences appear to be explained primarily by these tissue-related variables. Although men and women differ in muscle fiber type distribution (James et al., 2025) and in their susceptibility to adipose tissue fibrosis (Davis et al., 2013), these sex-specific tissue characteristics may not translate into meaningful differences in overall tissue stiffness when the limb is analyzed as a whole (Prado et al., 2005; Kovanen et al., 1984; Kielty et al., 2002).

From a practical perspective, these findings suggest that sex is not a major determinant of lower-limb AOP when limb composition is considered, which appears to be a more relevant predictor of occlusion pressure.

### 4.4 Limitations and future directions

The findings of this study suggest that individual lower-limb composition may be relevant to consider when determining arterial occlusion pressure to ensure accuracy and safety. However, several limitations should be acknowledged. The sample consisted of healthy young adults, which limits the generalizability of the findings to populations with different body composition profiles. Reliability values for AOP measurements could not be reported because the measure was recorded only once. The use of a single cuff model (18 cm) restricted statistical comparisons with other devices. The limb composition measures obtained by DXA reflect the composition of the entire limb rather than the specific region being compressed by the cuff. Site-specific measurements of the thigh could be more precise, given that this is the region where the cuff interacts with the involved tissues. Additionally, subcutaneous tissue thickness was not directly measured, and lean mass is not fully equivalent to skeletal muscle mass.

Future research should investigate these relationships in clinical and older populations with greater anthropometric variability, compare different cuff widths, and incorporate more precise methods for assessing limb composition. Longitudinal studies could also evaluate whether changes in lower-limb composition over time influence AOP, thereby contributing to more accurate long-term blood flow restriction training prescriptions.

## 5 Conclusion

The results of this study indicate that lower-limb composition is significantly associated with arterial occlusion pressure (AOP), particularly lower-limb lean mass and fat mass. However, these associations lose statistical significance when thigh circumference and systolic blood pressure (SBP) are accounted for. Additionally, thigh circumference demonstrated greater explanatory relevance for AOP in the seated and supine positions, whereas limb composition contributed more substantially in the standing position. Body position significantly modulated these relationships, with stronger associations observed in the standing condition. Sex was not a relevant predictor of AOP.

Taken together, these findings suggest that the relevance of limb composition for predicting AOP is posture-dependent and appears to be more pronounced in upright positions. However, in general, its influence seems smaller than that of established predictors, such as SBP and thigh circumference. Therefore, limb composition may be considered when using indirect methods—such as predictive equations or arbitrary pressures—in this position, potentially improving both the safety and effectiveness of blood flow restriction exercise prescription.

## Data Availability

The original contributions presented in the study are included in the article/**Supplementary Material**. Further inquiries can be directed to the corresponding authors.
