# Supplementary material for: Limb composition: implications for the determination of arterial occlusion pressure
Source: Front Physiol. 2026 May 29;17:1707774. doi: 10.3389/fphys.2026.1707774 (PMC13259813; doi:10.3389/fphys.2026.1707774)
Supplement: Supplementary file 1 [file Table1.docx]

**Table 1 -** Power analysis of the associations between limb composition and arterial occlusion pressure in different body positions.

| **Variable** | **Power** |
| --- | --- |
| **Supine position** | |
| Sex | 0.596 |
| Lean mass (kg) |  |
| Fat mass (kg) |  |
| **Seated position** | |
| Sex | 0.778 |
| Lean mass (kg) |  |
| Fat mass (kg) |  |
| **Standing position** | |
| Sex | 0.994 |
| Lean mass (kg) |  |
| Fat mass (kg) |  |

kg: kilogram

**Table 2 -** Power of the multivariable associations and relative contribution of limb composition, circumference, and systolic blood pressure to arterial occlusion pressure in different body positions.

| **Variable** | **Power** |
| --- | --- |
| **Supine position** | |
| Lean mass (kg) | 0.998 |
| Fat mass (kg) |  |
| Thigh Circumference |  |
| SBP |  |
| Sex |  |
| **Seated position** | |
| Lean mass (kg) | 0.999 |
| Fat mass (kg) |  |
| Thigh Circumference |  |
| SBP |  |
| Sex |  |
| **Standing position** | |
| Lean mass (kg) | 0.999 |
| Fat mass (kg) |  |
| Thigh Circumference |  |
| SBP |  |
| Sex |  |

kg: kilogram, SBP: systolic blood pressure

**Table 3 -** Power of the interactions between limb composition, body position, and sex in predicting arterial occlusion pressure**.**

| **Interaction** | **Power** |
| --- | --- |
| **Body position** | |
| Lean mass (kg) Standing x Seated position | 0.979 |
| Lean mass (kg) Standing x Supine position |  |
| Lean mass (kg) Seated x Supine position |  |
| Fat mass (kg) Standing x Seated position | 0.545 |
| Fat mass (kg) Standing x Supine position |  |
| Fat mass (kg) Seated x Supine position |  |
| **Sex** | |
| Lean mass (kg) x Sex | 0.116 |
| Fat mass (kg) x Sex | 0.070 |

kg: kilogram
